# A Case of Trapezium Avascular Necrosis Treated Conservatively

**DOI:** 10.1155/2017/6936013

**Published:** 2017-05-25

**Authors:** Evangelos Petsatodis, Konstantinos Ditsios, Panagiotis Konstantinou, Iosafat Pinto, Lazaros Kostretzis, Ioannis Theodoroudis, Mayia Pilavaki

**Affiliations:** ^1^Department of Radiology, G. Papanikolaou General Hospital, Thessaloniki, Greece; ^2^1st Department of Orthopaedics, G. Papanikolaou General Hospital, Aristotle University of Thessaloniki, Thessaloniki, Greece

## Abstract

**Introduction:**

Avascular necrosis (AVN) of the bones of the wrist most commonly involves the lunate followed by the proximal pole of the scaphoid and the capitate. Trapezium avascular necrosis is extremely rare with only two cases reported in the literature, both of which were treated surgically. In this article, we report a unique case of trapezium avascular necrosis treated conservatively.

**Case Presentation:**

A 38-year-old man complaining of a 4-month history of mild pain on the base of his right thumb. MRI scan was performed. The clinical presentation and the imaging findings indicated avascular osteonecrosis of the trapezium. The patient was treated with immobilization of the wrist joint for a period of six weeks. Three months later, the patient was free of symptoms and the MRI scan revealed a normal trapezium.

**Conclusion:**

AVN of trapezium is extremely rare. Our case shows that immobilization of an early stage avascular necrosis of the trapezium might be a treatment option.

## 1. Introduction

Avascular necrosis (AVN) is a disease where there is cellular necrosis of bone and marrow elements due to an interruption of the blood supply. It is a disease documented in several bones throughout the human body and most commonly affects the femoral head [1, 2].

AVN of the carpal bones is an uncommon condition that most commonly involves the lunate [3] and the scaphoid [4], followed by the capitate, pisiform, and trapezoid [5].

Trapezium AVN is extremely rare with only two cases reported in the literature, both of which were treated with bone excision and arthroplasty or vascularized bone graft [6, 7].

In our case, we report a trapezium AVN that was treated conservatively.

## 2. Case Presentation

A 38-year-old man was presented in the orthopaedic department of our hospital complaining of a 4-month history of mild pain on the base of the right thumb. The patient had no history of trauma, no systemic disease was reported, and he had not taken any corticosteroids. He is a white-collar bank worker and reported continuous mild pain that impaired his ability in everyday activities.

There was no inflammation and the mobility of the wrist and fingers was normal. Physical examination revealed tenderness over the trapezium without any swelling.

**Figure 1:**
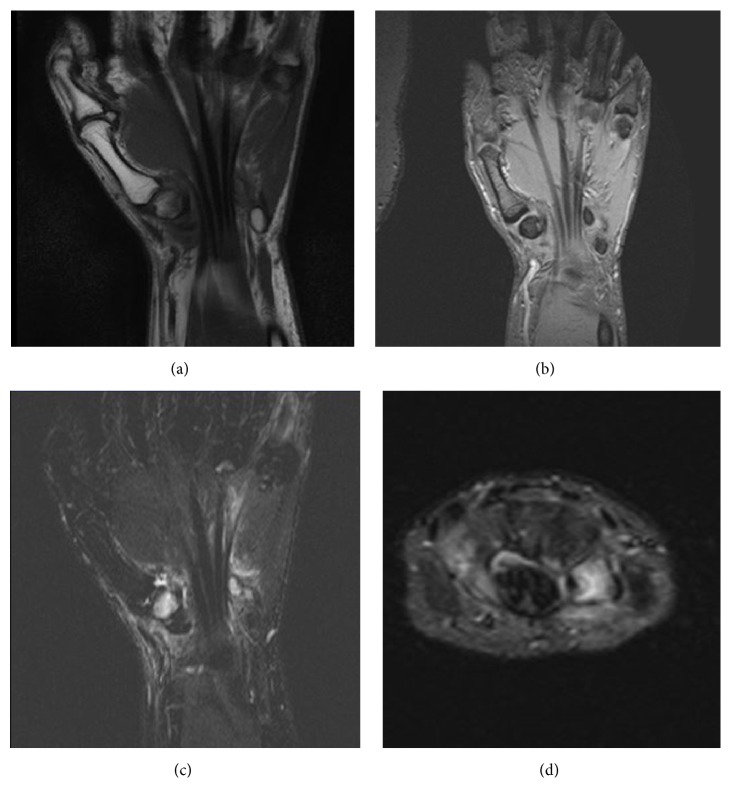
(a) T1-weighted coronal image reveals areas of low signal intensity affecting the trapezium. (b) PD coronal image shows heterogeneous signal intensity with areas of high signal intensity. ((c) and (d)) STIR coronal and axial images reveal increased signal intensity affecting the whole trapezium consistent with hyperemic areas due to repair process and oedema.

The patient underwent an X-ray examination which proved to be normal. Further investigation with an MRI of the wrist revealed an area of abnormal signal intensity affecting the whole trapezium. On T1-weighted MR images, the bone demonstrated low signal intensity while being hyperintense on PD and STIR sequences (Figure 1). The imaging findings were highly suggestive of early stage AVN of the trapezium.

Due to the mild symptoms, the age of the patient, and the blood supply of the trapezium, we decided to proceed with conservative treatment. The patient's wrist and thumb were immobilized with a thumb thermoplastic splint for six weeks and he was prescribed nonsteroidal anti-inflammatory drugs. Right after that period and because most of the symptoms were faded, he applied the splint only during the night for additional four weeks.

**Figure 2:**
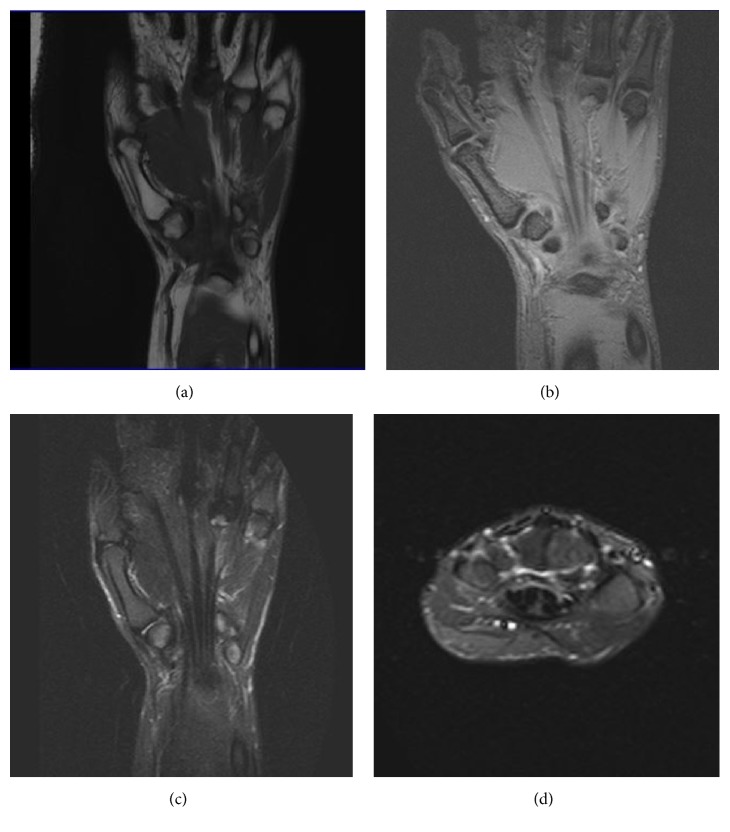
After three months, an MRI scan reveals an almost normal trapezium with patient's symptoms fully subsided. (a) TIWI coronal image. (b) PD coronal image. ((c) and (d)) STIR coronal and axial images.

After three months, the patient's symptoms subsided and an MRI scan revealed a normal trapezium (Figure 2).

## 3. Discussion

AVN is a condition defined as bone necrosis due to interruption of blood supply [8–10]. AVN of the wrist most commonly involves the lunate and the scaphoid. The major cause implicated in carpal bone AVN is trauma or microtrauma as in Kienbock disease [3, 8]. The mechanism of the disruption of the blood supply to the bone is mechanical vascular disruption, thrombosis and embolism, injury to a vessel, pressure on a vessel, or venous occlusion. Other conditions associated with AVN are long-term use of corticosteroids, alcohol, hemoglobinopathies, or systemic disorders (systemic lupus erythematosus, Gaucher's disease). In many cases, the cause cannot be identified.

The disruption of blood supply leads to bone oedema as seen on MRI scans.

Gelberman et al. described in detail the blood supply of the carpal bones [11–13]. The trapezium is supplied by two separate extraosseous systems. Radial artery branches supply directly the lateral, posterior, and palmar surfaces, while branches of the recurrent radial artery feed the palmar aspect. There are also intraosseous anastomoses between these two vessels. The trapezium together with the pisiform and the triquetrum is placed in group 3 which contains two or more areas of vessel entries and consistent intraosseous anastomoses. Due to this diffuse blood supply, the trapezium is considered as a very rare site of AVN development, in contrast to bones placed in groups 1 and 2, where there is a single-vessel or multivessel supply but scant intraosseous anastomoses [14]. This is the reason why group 1 bones (scaphoid, capitate, and 8% of the lunates) have a higher risk for developing AVN. AVN has also been described in group 2 bones, such as the trapezoid, after dislocation [15].

The role of MR imaging in the early diagnosis of AVN is well established. Findings include signal drop of the whole trapezium on T1WI and increased signal on T2WI and STIR images.

Its clinical presentation includes chronic pain, swelling, reduced mobility, and stiffness. An early diagnosis is vital due to the fact that it can lead to bone salvage if the correct treatment is applied.

The differential diagnosis of “mild” types of trapezium AVN should include bone oedema secondary to trauma, infection (however, in these cases, it is unlikely that the whole trapezium would be affected so homogeneously like in the presented patient), or basilar thumb arthritis, in which case the Χ-ray and MRI imaging would have been different. In our case, there was also no history of trauma or any clinical or laboratory findings of infection.

With this case report, the authors would like to emphasize the excellent results of conservative treatment (immobilization) in early stages of trapezium AVN, especially when the X-ray is diagnosed as “normal.” Actually, there is no specific classification for trapezium AVN as a result of the very few cases reported in the literature. We characterised this incident as a stage I AVN based on Steinberg et al. [16] and ARCO [17] classification for femoral head AVN. Possible treatment options for this type of AVN would be either conservative, such as immobilization and immobilization plus prostaglandins/teriparatide, or surgical, like bone decompression and excision arthroplasty, or vascularised bone graft as stated below. Surgery was the definitive treatment in the other two cases reported in the literature, although we decided, taking into consideration the age of the patient, his mild symptoms, and the diffuse blood supply of the trapezium, to continue with the conservative treatment.

There is no literature regarding trapezium AVN treated conservatively. However, there are articles documenting conservative treatment of early AVN stages, such as in hip, using teriparatide, alendronate [18], or prostaglandin I_2_ [19]. The immobilization as a conservative treatment combines the low cost therapy with no pharmacological side effects in contrast to prostaglandin or teriparatide therapy. Uneventful effects during prostaglandin therapy could be hypotension, arrhythmia, bleeding, thromboembolism, pulmonary oedema, allergic reactions, respiratory distress syndrome, flush headaches, and nausea [20], whereas the correlation between prolonged use of teriparatide and teriparatide-induced osteosarcoma is not clear yet.

To our knowledge, only two cases of AVN of the trapezium were reported so far and both of them were treated surgically either by suspension arthroplasty or by vascularized bone grafts [6, 7]. In the specific case, taking into consideration the mild symptoms of the patient and especially the diffuse blood supply of the trapezium, we decided to follow a conservative treatment. After three months, the clinical outcome of the patient was good and an MRI scan revealed a normal trapezium.

## 4. Conclusion

Avascular necrosis of the trapezium is extremely rare with only two cases reported in the literature so far, both of which were treated surgically. In our case, taking into consideration the diffuse blood supply of the trapezium, we decided to treat the patient conservatively resulting in a normal bone after three months. Conservative treatment should be considered as an option before surgery.
